# A Novel GNSS Interference Detection Method Based on Smoothed Pseudo-Wigner–Hough Transform

**DOI:** 10.3390/s21134306

**Published:** 2021-06-24

**Authors:** Kewen Sun, Baoguo Yu, Mireille Elhajj, Washington Yotto Ochieng, Tengteng Zhang, Jianlei Yang

**Affiliations:** 1 State Key Laboratory of Satellite Navigation System and Equipment Technology, Zhongshan West Road 589, Shijiazhuang 050081, China; yubg@sina.cn (B.Y.); yangjianlei555@163.com (J.Y.); 2 School of Computer & Information, Hefei University of Technology, Tunxi Road 193, Hefei 230009, China; 2019170914@mail.hfut.edu.cn; 3 Centre for Transport Studies, Imperial College London, London SW7 2AZ, UK; m.el-hajj11@imperial.ac.uk (M.E.); w.ochieng@imperial.ac.uk (W.Y.O.)

**Keywords:** interference detection, time-frequency (TF) analysis, cross-term, Hough transform (HT), smoothed pseudo-Wigner–Hough transform (SPWHT)

## Abstract

This paper develops novel Global Navigation Satellite System (GNSS) interference detection methods based on the Hough transform. These methods are realized by incorporating the Hough transform into three Time-Frequency distributions: Wigner–Ville distribution, pseudo -Wigner–Ville distribution and smoothed pseudo-Wigner–Ville distribution. This process results in the corresponding Wigner–Hough transform, pseudo-Wigner–Hough transform and smoothed pseudo-Wigner–Hough transform, which are used in GNSS interference detection to search for local Hough-transformed energy peak in a small limited area within the parameter space. The developed GNSS interference detection methods incorporate a novel concept of zero Hough-transformed energy distribution percentage to analyze the properties of energy concentration and cross-term suppression. The methods are tested with real GPS L1-C/A data collected in the presence of sweep interference. The test results show that the developed methods can deal with the cross-term problem with improved interference detection performance. In particular, the GNSS interference detection performance obtained with the smoothed pseudo-Wigner–Hough transform method is at least double that of the Wigner–Hough transform-based approach; the smoothed pseudo-Wigner–Hough transform-based GNSS interference detection method is improved at least 20% over the pseudo-Wigner–Hough transform-based technique in terms of the zero Hough-transformed energy percentage criteria. Therefore, the proposed smoothed pseudo-Wigner–Hough transform-based method is recommended in the interference detection for GNSS receivers, particularly in challenging electromagnetic environments.

## 1. Introduction

Complex electromagnetic environments affect satellite navigation applications. Although Global Navigation Satellite Systems (GNSS) are designed with a level of immunity from signal interference through the adoption of the direct-sequence spread spectrum (DSSS) scheme in signal design, the power of the received GNSS signal at the receiver antenna is very low [1]. Hence, unintentional or intentional disturbing signals have the potential to significantly degrade receiver performance [2,3]. Therefore, the problem of GNSS interference detection has become a major issue for researchers, industry and relevant governmental entities [4].

One of the most serious threats to GNSS receivers is radio frequency interference (RFI). This is because of the ease and simplicity of generating intentionally interfering signals using low-cost jamming devices. These jammers can radiate powerful and disruptive disturbing signals into the GNSS frequencies part of the L-band. The jammers usually emit mono- or multi-component nonstationary disturbing signals assumed to exhibit time-varying frequency characteristics. In addition, each GNSS interfering signal component is usually explained by a type of frequency or amplitude modulation. For example, linear frequency modulation (LFM) interference (i.e., linear chirp or sweep interference) is a relatively common and typical broadband nonstationary interference, which has the potential to seriously affect the performance of a GNSS receiver [2,3]. Moreover, unintentional GNSS interfering signals can result from faulty electronics (i.e., RFI sources co-existing with the GNSS signals), which are typically caused by the nonlinearities in the radio transmitters producing harmonics in the GNSS frequency bands. An example of unintentional interference is a continuous-wave RF signal originating from Digital Video Broadcast Terrestrial (DVB-T) services, which could present a serious threat to GNSS signals [5].

The linear frequency modulation or continuous-wave interference property usually results in a highly localizable power in the time-frequency (TF) plane. Therefore, time-frequency representations (TFRs) can be used in GNSS interference detection techniques [6,7,8,9,10,11,12,13,14]. It is known that the spectrogram has the unavoidable time-frequency resolution trade-off problem in accordance with the uncertainty principle, which shows impoverished time-frequency concentration properties [6,8,9,10,11]. Thus, the Wigner–Ville distribution (WVD) approach can be adopted in interference detection for GNSS receivers to overcome the time-frequency resolution limitation with the spectrogram technique [6,8,9,10,11]. Although the Wigner–Ville distribution approach can provide a near-optimal time-frequency resolution property among the time-frequency analysis-based techniques, it presents the serious and unacceptable cross interfering term problem resulting from the mutual interaction between different components of the corresponding signal [6,8,9,10,11]. In order to mitigate this problem, a type of kernel function can be adopted in Cohen’s class [15]. For example, the Choi–Williams transform (CWT) has been suggested to attenuate the influence of the cross-interfering term when detecting GNSS interfering signals [8]. In addition, reassignment methods have been proposed for the improvement of the time-frequency localization properties of the time-frequency analyses adopted in GNSS interference detection [9,10,16].

From another perspective, Wigner–Ville distribution transforms the analyzed signal into a time-frequency energy distribution in the time-frequency plane, whose shape depends on the frequency modulation law impressed on the analyzed signal [16]. When dealing with the sweep interference present in the received GNSS signal, a time-frequency distribution can be obtained with its energy focused on a straight line in the time-frequency domain by adopting Wigner–Ville distribution. Therefore, detecting sweep interference may be formulated as the problem of searching for a particular pattern of a linear form in the time-frequency domain. Furthermore, the detection of sweep interference can be completed by combining the Hough transform (HT) with the Wigner–Ville distribution, producing the Wigner–Hough transform (WHT) [17,18], which is a kind of straight-line integral projective transformation. In this way, Wigner–Hough transform can be adopted for the detection of linear patterns corresponding to the linear frequency modulation signal [19,20,21,22].

According to our knowledge, the Wigner–Hough transform was historically used for detecting GNSS interfering signals for the first time [23]. Hough transform-based techniques have played increasingly important roles in GNSS interference detection and have received more attention [24,25]; several reassigned time-frequency analyses have been used in combination with the Hough transform to construct improved interference detection methods for GNSS receivers [16,26], but the reassignment operations with these techniques require heavy computational complexities in the detection of GNSS interference. In addition, Hough transform-based techniques were also used in other navigation and radar-related applications [27,28].

Since pseudo-Wigner–Ville distribution (PWVD) is capable of suppressing the cross-interfering term in the quadratic time-frequency distributions by using a low-pass filtering window function [9,15], the pseudo-Wigner–Ville distribution has been combined with the Hough transform to obtain pseudo-Wigner–Hough transform (PWHT) to detect GNSS interference [25]. However, there are still residual cross-term components emerged in the parameter space, which limits the improvement of GNSS interference detection performance. Since smoothed pseudo-Wigner–Ville distribution (SPWVD) employs a low-pass filtering function in the two-dimensional form, the effect of the cross-interfering terms occurring between the multiple signal components can be effectively suppressed [9,11,15]; therefore, in order to break through the limitation of the pseudo-Wigner–Hough transform used in GNSS interference detection, in this paper, it’s reasonable to combine the smoothed pseudo-Wigner–Ville distribution with the Hough transform to propose a novel smoothed pseudo-Wigner–Hough transform (SPWHT) to further improve interference detection performance for GNSS receivers.

In order to analyze the properties of energy distribution concentration and cross-term suppression of the Hough transform-based techniques used in GNSS interference detection, a novel metric known as zero Hough-transformed energy percentage has been proposed in this paper. To the best of our knowledge, the proposed criteria of zero Hough-transformed energy percentage used for evaluating the Hough transform-based GNSS interference detection techniques are new.

We employ Wigner–Hough transform, pseudo-Wigner–Hough transform and smoothed pseudo-Wigner–Hough transform, respectively, for the detection of linear patterns corresponding to the type of sweep interference existing in the GNSS useful signal, and compare the performance of these Hough transform-based methods in GNSS interference detection. To verify the validity and effectiveness of these GNSS interference detection methods, we use real GPS L1-C/A data captured in an experimental scenario with a sweep interfering signal, which is a relatively common and typical nonstationary interference impeding the performance of GNSS receivers. The results show that the Hough transform-based techniques suppress the cross-interfering terms within the quadratic time-frequency distributions and improve GNSS interference detection performance compared with the traditional approaches based on time-frequency analyses. In particular, the zero Hough-transformed energy percentage analysis result shows that the interference detection performance obtained with the smoothed pseudo-Wigner–Hough transform method is at least double that of the Wigner–Hough transform approach; in contrast to the pseudo-Wigner–Hough transform-based technique, the zero Hough-transformed energy percentage obtained with the smoothed pseudo-Wigner–Hough transform-based GNSS interference detection method is improved at least 20% in comparison to the pseudo-Wigner–Hough transform-based technique. Therefore, the proposed novel smoothed pseudo-Wigner–Hough transform method is recommended in interference detection for GNSS receivers, particularly in challenging interfering environments.

## 2. GNSS Signal and Interference Models

In an interfering environment, the model of the signal at the input of a GNSS receiver is expressed as [8,9,10,11,16,25]:

(1) $$y_{RF}\left(t\right)=\overset{N_{s}}{\underset{i=1}{\sum }}r_{RF,i}\left(t\right)+\eta_{RF}\left(t\right)$$

where $r_{RF,i}\left(t\right)$ is the ith GNSS signal ($i=1,2,\;\cdots ,N_{s}$), $N_{s}$ denotes the number of satellites in view, and $\eta_{RF}\left(t\right)$ is the disturbing term. When a single useful signal is considered, for example, the GNSS signal emitted by the ith satellite can be expressed as [8,9,10,11,16]:

(2) $$r_{RF,i}\left(t\right)=A_{i}c_{i}\left(t-\tau_{i}\right)d_{i}\left(t-\tau_{i}\right)\cos\left[2\pi \left(f_{RF}+f_{d,i}\right)t+\varphi_{RF,i}\right]$$

where:

- $A_{i}$ is the amplitude of the ith useful GNSS satellite signal;
- $\tau_{i}$ is the propagation delay for the ith satellite signal;
- $c_{i}\left(t-\tau_{i}\right)$ denotes the Pseudo-Random Noise (PRN) code sequence extracted from a family of quasi-orthogonal codes modulated by rectangular pulses. For the simple case of the GPS L1-C/A signal, $c_{i}\left(t\right)$ represents the C/A code;
- $d_{i}\left(t-\tau_{i}\right)$ represents the navigation data bit information;
- $f_{RF}$ denotes the carrier center frequency of the GNSS signal, for the GPS L1-C/A signal, $f_{RF}=f_{L1}=$ 1575.42 MHz;
- $f_{d,i}$ denotes the Doppler frequency shift affecting the ith useful GNSS signal;
- $\varphi_{RF,i}$ is the initial carrier phase offset for the ith GNSS useful signal.

The disturbing term $\eta_{RF}\left(t\right)$ can be written as [8,9,10,11,16,25]:

(3) $$\eta_{RF}\left(t\right)=j_{RF}\left(t\right)+w_{RF}\left(t\right)$$

where $j_{RF}\left(t\right)$ is the nonstationary interfering signal, and $w_{RF}\left(t\right)$ is the GNSS receiver thermal noise, usually in the form of a zero-mean stationary additive white Gaussian noise (AWGN) process.

There are different forms of interfering signals that could be generated by potential sources of radio frequency interference. In this paper, without loss of generality, the interference term $j_{RF}\left(t\right)$ can be considered as the sweep interference (i.e., linear chirp), which is usually linearly frequency modulated with an almost constant amplitude and typically regarded as a test bench in interference detection for GNSS applications.

Sweep interference, one of the main types of the interfering signals, can be expressed by sinusoids in the time domain as [16]:

(4) $$j_{RF}\left(t\right)=A_{inst}\left(t\right)\cos\left[2\pi \int_{0}^{t}f_{inst}\left(t\right)dt+\varphi_{0}\right]$$

where $A_{inst}\left(t\right)$ is the carrier amplitude of the sweep interfering signal, $f_{inst}\left(t\right)$ represents the instantaneous frequency of the sweep interference, and $\varphi_{0}$ denotes the initial carrier phase of the sweep interference, which can be considered as a random variable presenting a uniform distribution in the range [−π,+π).

When considering a linear chirp, its instantaneous frequency $f_{inst}\left(t\right)$ linearly evolves with time in the interval $\left[f_{RF}+\Delta f_{0},\;f_{RF}+\Delta f_{1}\right]$, where $f_{RF}$ is the center frequency of the considered GNSS signal. In this way, the instantaneous frequency $f_{inst}\left(t\right)$ of the linear chirp can be written as [16,25]:

(5) $$f_{inst}\left(t\right)=f_{0}+kt\;\;\;\;\;\;\;\;0\leq t\leq t_{j}$$

where $f_{0}$ is the initial frequency, i.e., $f_{0}=f_{RF}+\Delta f_{0}$, $t_{j}$ denotes the frequency sweep period of the interfering signal, and k is the chirp rate or the frequency increase rate, expressed as [16,25]:

(6) $$k=\frac{f_{1}-f_{0}}{t_{j}}=\frac{\Delta f_{1}-\Delta f_{0}}{t_{j}}$$

where $f_{1}$ is the final frequency in the frequency sweep period $t_{j}$, i.e., $f_{1}=f_{RF}+\Delta f_{1}$, and $f_{1}-f_{0}=\Delta f_{1}-\Delta f_{0}$ is the value of the frequency sweep.

The input signal $y_{RF}\left(t\right)$ defined in Equation (1) is filtered, and down-converted by the GNSS receiver front-end. Then, the received GNSS signal before the Analog to Digital (A/D) conversion can be written as [8,9,10,11,16,25]:

(7) $$\begin{array}{ll} y\left(t\right) & =\overset{N_{s}}{\underset{i=1}{\sum }}r_{i}\left(t\right)+\eta \left(t\right)\;\\ & =\overset{N_{s}}{\underset{i=1}{\sum }}A_{i}{\tilde{c}}_{i}\left(t-\tau_{i}\right)d_{i}\left(t-\tau_{i}\right)\cos\left[2\pi \left(f_{IF}+f_{d,i}\right)+\varphi_{i}\right]+\eta \left(t\right) \end{array}$$

where $f_{IF}$ is the IF of the GNSS receiver, and ${\tilde{c}}_{i}\left(t-\tau_{i}\right)$ is the spreading code sequence after filtering of the GNSS receiver front-end. Here, the effect of the GNSS receiver front-end filter is neglected, assuming the simplifying condition ${\tilde{c}}_{i}\left(t\right)\approx c_{i}\left(t\right)$; η(t) is the disturbing component after down-conversion and filtering, η[t]=j[t]+ω[t].

## 3. Time-Frequency Transforms

The common and simple technique used for evaluating the time-varying signal frequency characteristic is short-time Fourier transform (STFT) [15,29]. In the simple case for the continuous-time nonstationary signal, short-time Fourier transform can be represented as [10,15,29]:

(8) $$STFT\left(t,\omega \right)=\int_{-\infty }^{+\infty }y_{a}\left(\tau \right)h\left(\tau -t\right)e^{-j\omega t}d\tau$$

where h(t) is the analysis window function, which is typically real and even and centered around zero, $y_{a}\left(t\right)$ represents the analytic signal to be analyzed, and STFT(t,ω) is a linear function depending on the selected window function h.

The spectrogram can be defined as the squared magnitude of short-time Fourier transform, expressed as [10,15,29]:

(9) $$SPEC\left(t,\omega \right)={\left|STFT\left(t,\omega \right)\right|}^{2}$$

**Figure 1:**
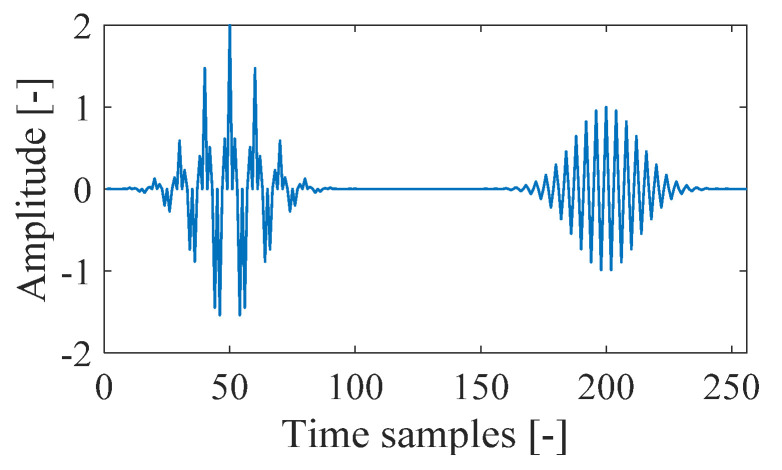
The nonstationary signal composed of three transient Gaussian components.

**Figure 2:**
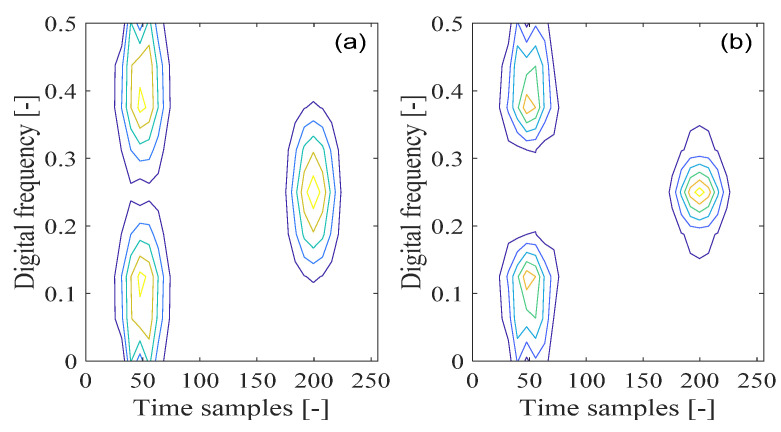
The spectrogram of nonstationary signal composed of three transient Gaussian components; (**a**) Chebyshev window; (**b**) Kaiser window.

The nonstationary signal, which consists of three transient Gaussian components with different positions in time and frequency, is shown in Figure 1. By changing the types of analysis window functions, the spectrogram of the signal is shown in Figure 2. In Figure 2a, the Chebyshev window is selected, and in Figure 2b, the Kaiser window is chosen, and the window length is set to 15 samples in both cases. From Figure 2, it can be observed that the spectrogram shows different time-frequency resolutions based on the selected type of window function, which usually provides insufficient properties of the time-frequency resolution.

**Figure 3:**
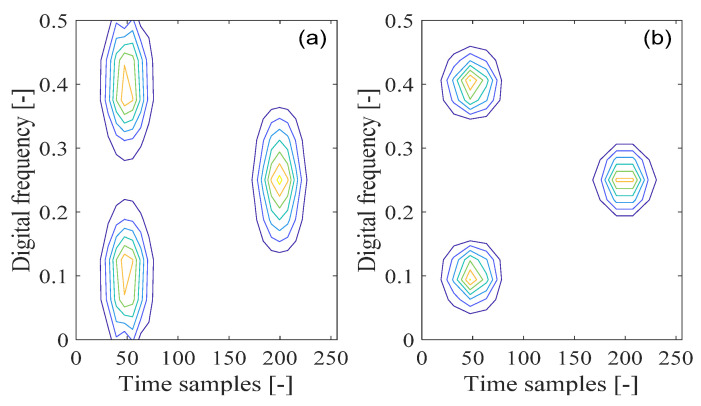
The spectrogram of a nonstationary signal composed of three transient Gaussian components by adopting the Hamming type of window function; (**a**) 15 samples window length; (**b**) 31 samples window length.

Furthermore, in order to determine the time-frequency resolution characteristics of the spectrogram by varying the window function size in the time-frequency analyses, the spectrogram of the aforementioned signal is shown in Figure 3, where the Hamming type of the window function is adopted. In Figure 3a, the window length is set to 15 samples. It can be seen that the small size of the analysis window function in the time domain provides impoverished resolution in the frequency domain. From Figure 3b, when the analysis window length is set to 31 samples, it is shown that increasing the window size results in an increased resolution in the frequency domain. In this way, the limitation from the uncertainty principle with the spectrogram can be further verified, pointing to the trade-off problem of time-frequency resolution due to the adoption of the analysis window function in the spectrogram.

The commonly used solution for the spectrogram’s time-frequency resolution trade-off problem is to adopt Wigner–Ville distribution in the time-frequency analysis, which can be explained as [8,9,10,11,15,29]:

(10) $$WVD\left(t,\omega \right)=\frac{1}{2\pi }\int_{-\infty }^{+\infty }R\left(t,\tau \right)e^{-j\omega \tau }d\tau =\frac{1}{2\pi }\int_{-\infty }^{+\infty }y_{a}(t+\frac{\tau }{2})y_{a}^{*}\left(t-\frac{\tau }{2}\right)e^{-j\omega \tau }d\tau$$

where τ is the lag variable, $R\left(t,\tau \right)=y_{a}\left(t+\frac{\tau }{2}\right)y_{a}^{*}\left(t-\frac{\tau }{2}\right)$ is the instantaneous auto-correlation function, and (∗) is the operation of complex conjugate.

**Figure 4:**
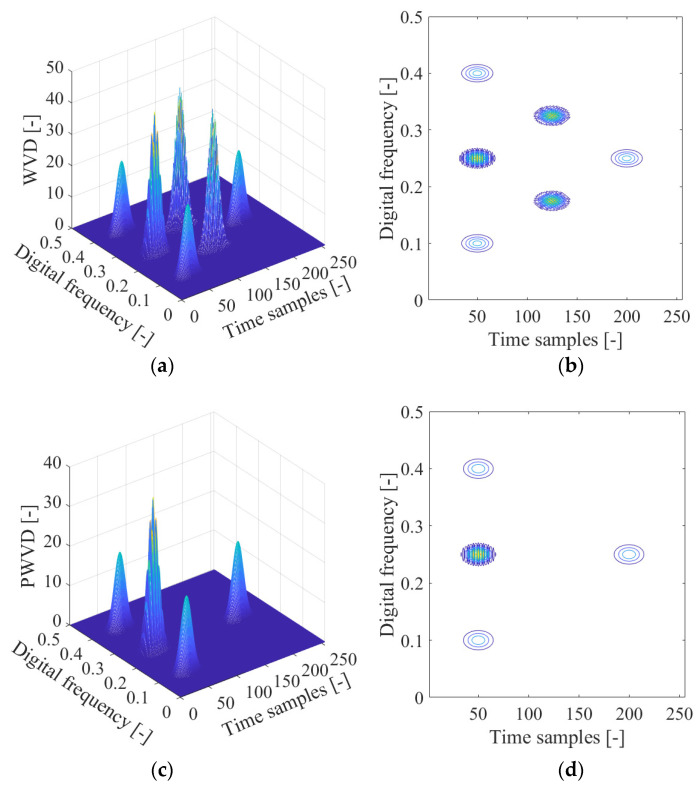

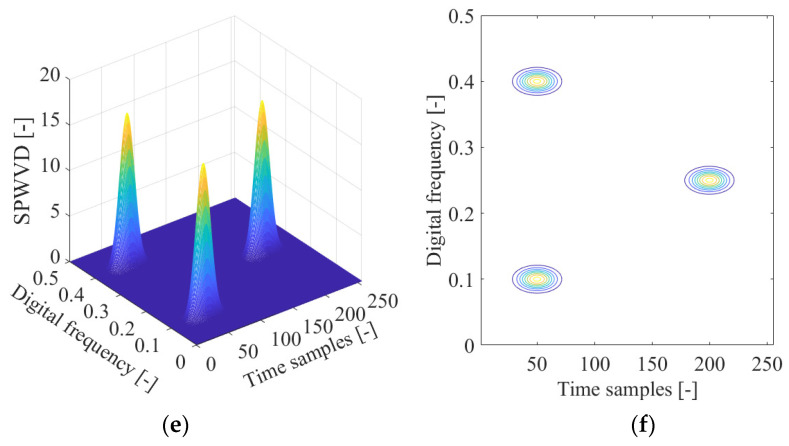
The time-frequency distributions of a nonstationary signal composed of three transient Gaussian components. (**a**) Wigner–Ville distribution (WVD); (**b**) WVD’s contour; (**c**) Pseudo-Wigner–Ville distribution (PWVD); (**d**) PWVD’s contour; (**e**) Smoothed pseudo-Wigner–Ville distribution (SPWVD); (**f**) SPWVD’s contour.

The Wigner–Ville distribution presents several desirable properties and provides a near-optimal time-frequency resolution among all the time-frequency distributions. However, its main drawback comes from undesirable cross-term interference [6,8,9,10,11,15,29]. An example of the Wigner–Ville distribution of the nonstationary signal is shown in Figure 4a, and the corresponding contour of the calculated Wigner–Ville distribution is shown in Figure 4b. It can be seen from Figure 4a that there are three time-frequency energy peaks, which represent the corresponding auto-terms of the analyzed signal in the time-frequency domain. It is also visible that the Wigner–Ville distribution exhibits improved time-frequency resolution properties over the spectrogram. From Figure 4b, it can be seen that three cross interfering terms emerge among the transient Gaussian components separated between different positions in the time or frequency domains within the time-frequency plane. It is also clear that the magnitudes corresponding to the oscillatory cross interfering terms are twice as large as those of the related auto-terms of the analyzed signal. Although a number of time-frequency desirable properties can be obtained with the Wigner–Ville distribution, due to its bilinear form of the time-frequency distribution, the distribution inevitably presents serious cross interfering terms in the time-frequency domain. The presence of the cross-interfering terms in the time-frequency plane does not possess any physical meaning, making signal understanding and correct interpretation near impossible.

A reasonable approach to partially mitigate the influence of cross-interfering terms is to introduce a low-pass filtering window function in the Wigner–Ville distribution, resulting in the concept of pseudo-Wigner–Ville distribution [9,11,15,29]. We know that the Wigner–Ville distribution approach applies equal weights temporally; therefore, if we have an interest in concentrating on the signal near a given time instant t, then we can multiply the instantaneous auto-correlation function $y_{a}\left(t+\frac{\tau }{2}\right)y_{a}^{*}\left(t-\frac{\tau }{2}\right)$ by an analysis window function h(τ). Thus, the pseudo-Wigner–Ville distribution can be obtained. The pseudo-Wigner–Ville distribution can be explained as the common Wigner–Ville distribution with a window function in the direction of time, which can be expressed as [9,11,15,29]:

(11) $$PWVD\left(t,\omega \right)=\frac{1}{2\pi }\int_{-\infty }^{+\infty }h\left(\tau \right)y_{a}\left(t+\frac{\tau }{2}\right)y_{a}^{*}\left(t-\frac{\tau }{2}\right)e^{-j\omega \tau }d\tau$$

where the window function h(τ) can be used to control PWVD(t,ω), which usually acts as an even function and presents its peak value around the time instant τ=0.

In the pseudo-Wigner–Ville distribution method, the adoption of the analysis window function makes its distribution more local in the time-frequency domain. This is beneficial for the suppression of the cross-interfering terms for the analyzed signal with multi-components. The time-windowing operation can be considered to be equal to the frequency filtering applied in the Wigner–Ville distribution approach. In this way, the corresponding cross interfering term localized among the time-shifted signal components can be alleviated to some extent. For example, the calculated pseudo-Wigner–Ville distribution for the analyzed signal is shown in Figure 4c with the corresponding contour presented in Figure 4d. It can be seen that the cross-term between the pair of auto-terms located in different time positions is effectively mitigated. This is in contrast to the cross-term between the pair of auto-terms distributed in the same time position, which still exists in the time-frequency domain.

The cross-interfering term localized among the signal components with the same time position in the time-frequency plane can be further suppressed by adopting the smoothing window function with the two-dimensional form expressed in the directions of both time and frequency. In this case, smoothed pseudo-Wigner–Ville distribution, i.e., the smoothed version of pseudo-Wigner–Ville distribution, can be obtained as [9,11,15,29]:

(12) $$SPWVD\left(t,\omega \right)=\frac{1}{2\pi }\int_{-\infty }^{+\infty }\int_{-\infty }^{+\infty }g\left(u\right)h\left(\tau \right)y_{a}\left(t+u+\frac{\tau }{2}\right)y_{a}^{*}\left(t+u-\frac{\tau }{2}\right)e^{-j\omega \tau }d\tau du$$

where h denotes the lag window function, and g represents the time smooth window function.

For example, the smoothed pseudo-Wigner–Ville distribution of the analyzed signal is shown in Figure 4e with the corresponding contour presented in Figure 4f. It can be seen that there are only three auto-terms remaining correctly in the time-frequency plane, which represent the corresponding signal components. It can be known that the cross-term with the pseudo-Wigner–Ville distribution between the pair of auto-terms located in the same time position is removed due to the use of low-pass filtering in the two-dimensional form within the smoothed pseudo-Wigner–Ville distribution; this further confirms the benefit of the smoothed pseudo-Wigner–Ville distribution method in suppressing the cross-term over the Wigner–Ville distribution and pseudo-Wigner–Ville distribution approaches. On the other hand, it can be observed that the resulting time-frequency resolution properties of the auto-terms for the multi-component signal are degraded compared with the Wigner–Ville distribution approach. Therefore, in the smoothed pseudo-Wigner–Ville distribution, the time-window overlay operation can be adopted to effectively mitigate the cross-interfering terms among different signal components; however, this benefit is accrued at the price of a deterioration of the signal’s auto-terms in the time-frequency domain.

## 4. Smoothed Pseudo-Wigner–Hough Transform

The Hough transform is commonly used to isolate features of a particular shape within an image space, which can be particularly employed to change a difficult global detection of features in the image space into a relatively easy problem of local peak detection in the parameter space. In the parameter space, the noisy input points are usually randomly distributed, which cannot be effectively accumulated; therefore, the accumulated energy is fairly weak. On the other hand, in the image space, all the collinear points on the same straight line representing the frequency modulation law of the analyzed signal indicate their effective contributions to the feature extraction by accumulating the corresponding energy peak concentrated in the parameter space.

**Figure 5:**
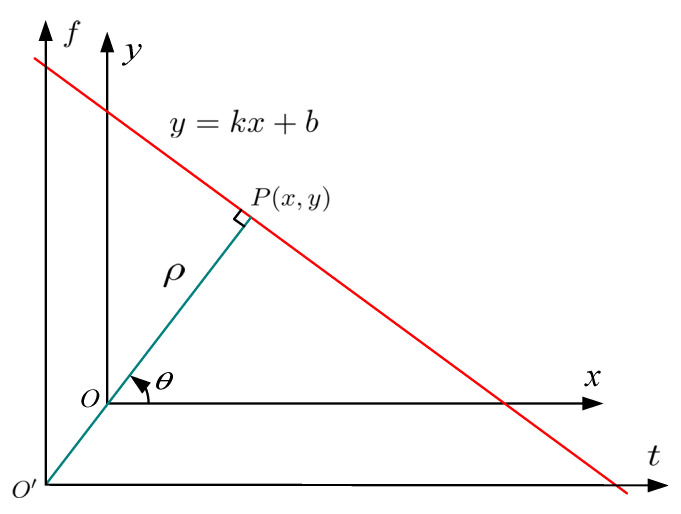
The normal parameterization of a straight line.

The simple case of the Hough transform can be adopted to detect the straight lines; it is known that the straight line y=kx+b can be generally expressed as a point (b,k) in the parameter domain; however, there are problems for the cases of the vertical lines which result in unbounded values of the slope parameter k. Therefore, in Figure 5, the radius-and-angle parametrization of a straight line is employed due to computational complexity reasons, which can be defined algebraically by the following normal notion [25]:

(13) ρ=xcosθ+ysinθ

where a point P(x,y) in the x−y image space can be mapped as (ρ,θ) in a ρ−θ transform space, ρ is the length of a normal from the coordinate origin to the straight line, and θ represents the orientation of ρ with respect to the x axis.

In this way, it is reasonable to associate a pair (ρ,θ) in the polar Hough parameter space with each straight line in the Cartesian image space. If a single point (x,y) is considered in the Cartesian image space, then the family of all the straight lines that pass through this point (x,y) definitely maps to a particular curve with the sinusoidal form in the ρ−θ polar Hough parameter space; furthermore, this mapping relationship is deterministic and unique. A set of two or more points that connect a straight line in the cartesian image space is bound to generate a series of sinusoidal curves that all intersect at the same point (ρ,θ) in the transform space corresponding to the determined straight line. Therefore, the problem of globally searching for collinear points on a straight line in the Cartesian image space is able to be effectively converted to the easily solved problem of detecting concurrent sinusoidal curves locally intersected in the same point (ρ,θ) within the polar Hough parameter space. Thus, an interesting and effective scheme for detecting straight lines can make use of this point-line transformation. The detection of the point (ρ,θ) in the polar transform space is relatively easier and more effective than detecting multiple collinear points in the Cartesian image space. When the position of the intersection point (ρ,θ) of concurrent sinusoidal curves is determined by searching for the Hough-transformed energy peak in the ρ−θ transform space, the parameters of the corresponding straight line in the Cartesian image space can then be estimated [16].

As an efficient solution to search for straight lines in the image space, the Hough transform can be used in combination with the Wigner–Ville distribution to develop the Wigner–Hough transform method [17,18], which can be used to deal with the cross-term problem with the Wigner–Ville distribution; hence, the Wigner–Hough transform can be used in GNSS interference detection [23]. When considering the GNSS signal y(t), the Wigner–Hough transform can generally be explained as the mapping from the time-frequency domain to the parameter domain (f,k), which can be realized by the integral transform, expressed as [16,25]:

(14) $$WHT\left(f,k\right)=\int_{-\infty }^{+\infty }\int_{-\infty }^{+\infty }y_{a}\left(t+\frac{\tau }{2}\right)y_{a}^{*}\left(t-\frac{\tau }{2}\right)e^{-j2\pi \left(f+kt\right)\tau }d\tau dt$$

where $y_{a}\left(t\right)$ represents the analytic form of the signal y(t) to be analyzed, and f and k are the initial frequency and chirp rate for the analyzed signal, respectively.

By using the form of polar coordinates, Wigner–Hough transform in Equation (14) can be rewritten as [25]:

(15) $$WHT\left(\rho ,\theta \right)=\int_{-\infty }^{+\infty }\int_{-\infty }^{+\infty }y_{a}\left(t+\frac{\tau }{2}\right)y_{a}^{*}\left(t-\frac{\tau }{2}\right)e^{-j2\pi \frac{1}{\sin\theta }\left(\rho -\cos\theta \cdot t\right)\tau }d\tau dt$$

where θ ranges in the interval [0,π).

The Wigner–Hough transform may also be explained as a line integral of Wigner–Ville distribution, expressed as [25]:

(16) $$\begin{array}{ll} WHT\left(f,k\right) & =\int_{-\infty }^{+\infty }\int_{-\infty }^{+\infty }WVD\left(t,v\right)\delta \left(v-f-kt\right)dvdt\\ & =\int_{-\infty }^{+\infty }WVD\left(t,f+kt\right)\mathrm{dt}\;\\ & =\int_{-\infty }^{+\infty }WVD(t,\frac{1}{\sin\theta }\left(\rho -\cos\theta \cdot t\right))dt \end{array}$$

where δ(·) is the Dirac delta function.

The pseudo-Wigner–Ville distribution has been introduced to partially suppress the cross-interfering terms with the Wigner–Ville distribution [9,15], as can been verified in Section 3; the Hough transform can be further combined with the pseudo-Wigner–Ville distribution to develop the pseudo-Wigner–Hough transform to improve GNSS interference detection performance [25]. The definition of pseudo-Wigner–Hough transform is given as:

(17) $$PWHT\left(f,k\right)=\int_{-\infty }^{+\infty }\int_{-\infty }^{+\infty }h\left(\tau \right)y_{a}\left(t+\frac{\tau }{2}\right)y_{a}^{*}\left(t-\frac{\tau }{2}\right)e^{-j2\pi \left(f+kt\right)\tau }d\tau dt$$

Similar to the Wigner–Hough transform, the pseudo-Wigner–Hough transform in Equation (17) can also be explained as the line integral of pseudo-Wigner–Ville distribution, expressed as:

(18) $$PWHT\left(f,k\right)=\int_{-\infty }^{+\infty }\int_{-\infty }^{+\infty }PWVD\left(t,v\right)\delta \left(v-f-kt\right)dvdt\;=\int_{-\infty }^{+\infty }PWVD\left(t,f+kt\right)dt\;\;=\int_{-\infty }^{+\infty }PWVD(t,\frac{1}{\sin\theta }\left(\rho -\cos\theta \cdot t\right))dt$$

From Section 3, it is shown that smoothed pseudo-Wigner–Ville distribution can be used to mitigate the cross-interfering terms with the bilinear time-frequency distribution. However, this is achieved at the cost of a deteriorating time-frequency localization property [9]. Therefore, in this paper, the smoothed pseudo-Wigner–Ville distribution can be further combined with the Hough transform to propose a novel smoothed pseudo-Wigner–Ville distribution-Hough transform (i.e., smoothed pseudo-Wigner–Hough transform, SPWHT) in interference detection for GNSS receivers. The smoothed pseudo-Wigner–Hough transform can be then defined as:

(19) $$SPWHT\left(f,k\right)=\int_{-\infty }^{+\infty }\int_{-\infty }^{+\infty }\int_{-\infty }^{+\infty }g\left(u\right)h\left(\tau \right)y_{a}\left(t+u+\frac{\tau }{2}\right)y_{a}^{*}\left(t+u-\frac{\tau }{2}\right)e^{-j2\pi \left(f+kt\right)\tau }d\tau dudt$$

Moreover, the ρ−θ transform space should be discrete for the Hough transform based GNSS interference detection methods.

## 5. Hough-Transformed Energy Distribution Evaluation Scheme

**Figure 6:**
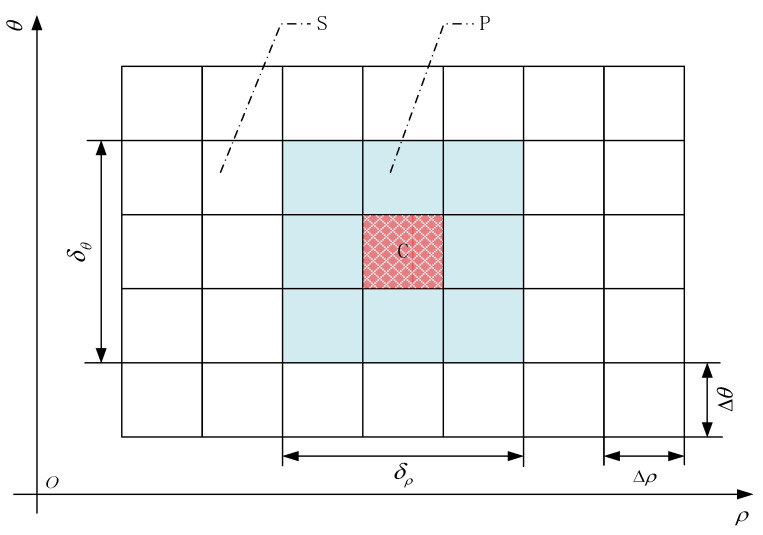
The zero Hough-transformed energy distribution evaluation scheme for the Hough transform-based GNSS interference detection techniques.

In order to understand the Hough-transformed energy distribution of the interfered GNSS signal in the ρ−θ parameter space, in this paper, a metric known as zero Hough-transformed energy percentage is proposed to evaluate the properties of energy distribution concentration and cross-term suppression in the GNSS interference detection. The concept of zero Hough-transformed energy percentage is explained in Figure 6. The ρ−θ parameter space S is a grid of cells where the Hough-transformed energy distribution is evaluated. The grid is with the two dimensions of ρ and θ; Δρ is the cell size in the direction of the ρ axis, and Δθ is the cell size in the direction of the θ axis.

Assuming that the peak of Hough-transformed energy distribution is located in a specific cell C within the parameter space S, and then the cell C can be selected as the geometric center to construct a rectangular local neighborhood domain P, where the main part of the Hough-transformed energy is concentrated; $\delta_{\rho }$ is the size of the rectangular local neighborhood region P in the direction of the ρ axis, and $\delta_{\theta }$ is the size of the rectangular local neighborhood region P in the direction of the θ axis. In this way, the entire parameter space S is divided into two parts: the local neighborhood domain P, which dominates most of the Hough-transformed energy, and the other remaining area (S−P), where the residual Hough-transformed energy is contained.

In order to compare the properties of energy concentration and cross-term suppression among the Wigner–Hough transform, the pseudo-Wigner–Hough transform, and the smoothed pseudo-Wigner–Hough transform techniques used in GNSS interference detection, the concept of zero Hough-transformed energy percentage can be explained as:

(20) $$\alpha =\frac{N_{zero}}{N}\times 100\%$$

where $N_{zero}$ is the number of cells in the region (S−P), where the values of the Hough-transformed energy distribution are equal to zero, and N is the total number of cells contained in the (S−P) area within the parameter space S, which excludes the local neighborhood area P.

For any type of Hough-transformed technique used in GNSS interference detection, when the zero Hough-transformed energy percentage α is larger, the Hough-transformed energy distribution is more concentrated in the intersection point of concurrent sinusoidal curves in the parameter space, and the residual Hough-transformed energy is effectively suppressed to a lower level even equal to zero in the other area (S−P) except the local intersection domain P, indicating that the cross-terms are more significantly mitigated.

## 6. Performance Evaluation

The performances of the proposed GNSS interference detection methods based on the Hough transform are comprehensively analyzed in this section. The proposed GNSS interference detection methods are compared to the traditional time-frequency analysis-based techniques such as spectrogram and Wigner–Ville distribution. Then, according to the zero Hough-transformed energy distribution percentage criteria, the performances of GNSS interference detection are further analyzed and compared among the proposed Hough transform-based techniques.

**Figure 7:**
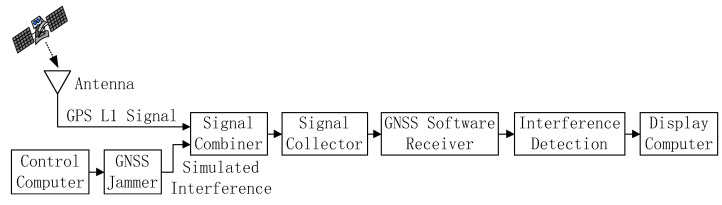
The scheme of the GNSS interference detection experiment.

**Figure 8:**
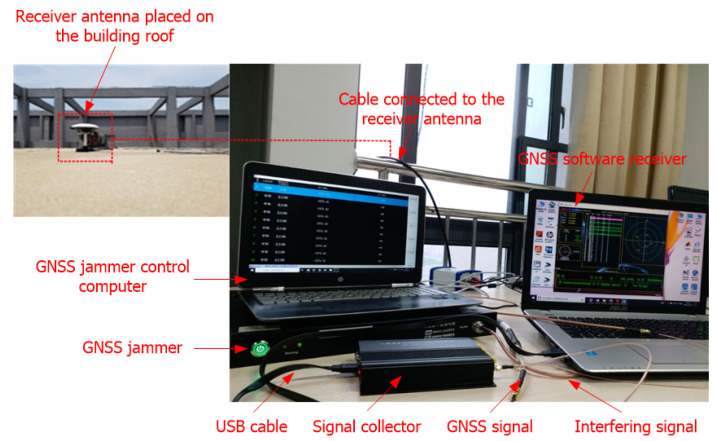
The experimental platform of the GNSS interference detection test.

The scheme of the GNSS interference detection experiment is shown in Figure 7. In the GNSS interference detection experiment, a GNSS signal collector was used to collect GPS L1-C/A signal through a cable connected to a GNSS receiver antenna placed on the roof of the Feicui Building at Feicui Lake Campus, Hefei University of Technology, China. At the same time, a GNSS jammer controlled by a computer was used to generate a sweep interfering signal, which was simultaneously added to the real GPS L1-C/A signal through a signal combiner. Then, the combined GPS L1-C/A signal and sweep interference were collected by a signal collector, which was sent to a GNSS software receiver implemented on a display computer through a USB cable. The adopted experimental platform of the GNSS interference detection test is illustrated in Figure 8.

The experimental scenario used in the GNSS interference detection test is characterized by the setup parameters shown in Table 1, which denote the collected GPS L1-C/A signal in zero-mean white Gaussian noise disturbed by a constant amplitude linear frequency modulation interference (i.e., linear chirp). For the experimental setup parameters, the carrier power-to-noise density ratio (C/N_0_) of the GPS L1-C/A signal is 46 dB-Hz; the jammer-to-noise ratio (JNR) of the linear chirp is −6 dB; and the instantaneous frequency $f_{inst}$ of the sweep interference linearly evolves with the time in the interval $\left[f_{L1}+\Delta f_{0},f_{L1}+\Delta f_{1}\right]$, where $f_{L1}$ is the center frequency of the GPS L1-C/A signal, $\Delta f_{0}$ is 6 MHz and $\Delta f_{1}$ is −9 MHz.

**Figure 9:**
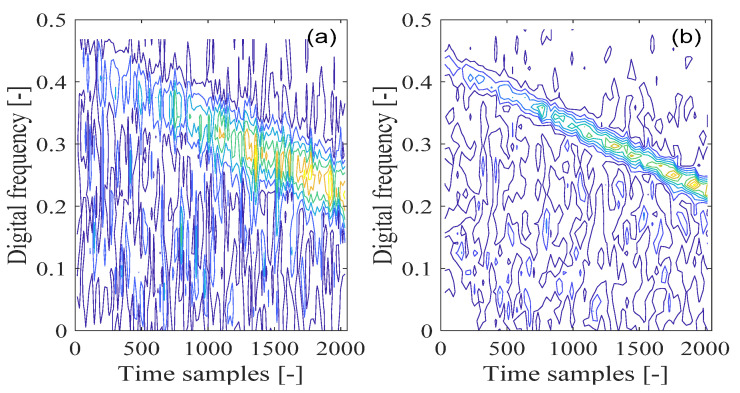
The spectrogram of GPS L1-C/A signal disturbed by the sweep interference; (**a**) window size = 31; (**b**) window size = 63.

Figure 9 depicts the spectrogram of the GPS L1-C/A signal in the presence of the sweep interference, and the Hamming type of the analysis window function is selected in the spectrogram analysis. In Figure 9a, the analysis window length is 31 samples. Although the sweep interference effect can be observed, it is impossible to correctly detect the sweep interference in the time-frequency plane since the spectrogram provides an insufficient time-frequency resolution. To analyze the time-frequency characteristics of the spectrogram approach by increasing the analysis window size, the spectrogram of the GPS L1-C/A signal in the presence of sweep interference is provided in Figure 9b, where the analysis window size is set to 63 samples; in this case, although the linear frequency modulation interference present in the received GPS L1-C/A signal can be more or less identified in the time-frequency plane, unsatisfactory time-frequency localization property in the time domain is obtained with the increased window width by using the spectrogram. Therefore, the spectrogram is not able to be practically used to provide a precise estimation of the characteristic parameters for the GNSS interfering signal, although an approximate evaluation can be made.

**Figure 10:**
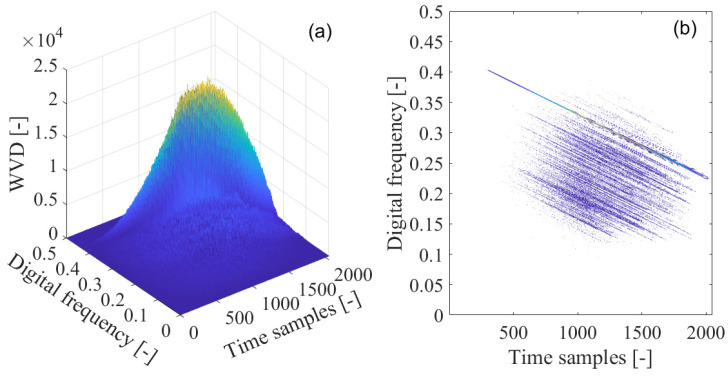
The Wigner–Ville distribution (WVD) of the GPS L1-C/A signal disturbed by the sweep interference; (**a**) WVD; (**b**) WVD’s contour.

Figure 10a depicts the Wigner–Ville distribution of the GPS L1-C/A signal in the presence of the sweep interference, with the corresponding contour of the calculated Wigner–Ville distribution in Figure 10b. From Figure 10, the sweep interference component shows a linear behavior in the time-frequency plane, which is located in the limited linear area of the time-frequency space. In particular, in Figure 10b, a straight line of the frequency modulation law corresponding to the sweep interference present in the received GNSS signal can be clearly observed in the time-frequency plane. However, this improved time-frequency resolution is at the price of the unavoidable cross-terms present in the time-frequency plane, which bring much error to the characteristic parameters’ estimation of the GNSS interfering signal and make signal interpretation difficult and incorrect. Due to the presence of a cross-term problem in the Wigner–Ville distribution, the GNSS interference essential features cannot be correctly extracted.

**Figure 11:**
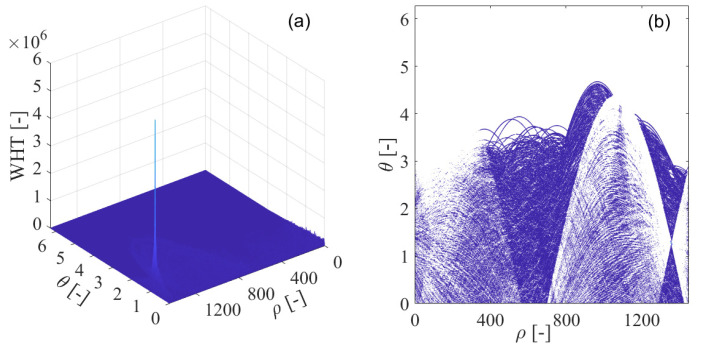
The Wigner–Hough transform (WHT) of the GPS L1-C/A signal disturbed by sweep interference; (**a**) WHT; (**b**) WHT’s contour.

In order to solve the problem of the cross-term with the Wigner–Ville distribution, Wigner–Hough transform is employed in the interference detection test for GNSS receivers, which is illustrated in Figure 11. In Figure 11a, the Wigner–Hough transform of the interfered GNSS signal is given in the ρ−θ plane. From Figure 11b, it is clear that the sinusoids corresponding to the white Gaussian noise or GNSS useful signal components are randomly distributed and cannot be accumulated in effect. On the contrary, the energy peak of Wigner–Hough transform is evidently located in the parameter space, which represents the sweep interfering signal occurring in the received GNSS signal. Therefore, the GNSS interference feature can be distinguished from those of the white Gaussian noise or the useful GNSS signal.

In Figure 11b, the contour of the computed Wigner–Hough transform is shown, where concurrent sinusoids pass through a specific single point in the ρ−θ transform space, with each sinusoidal curve corresponding to a single point on the line of GNSS sweep interference frequency modulation law in the time-frequency plane. In this way, in the time-frequency plane, the detection of the straight line of GNSS interference frequency modulation law has been converted to the equivalent determination of the position of the intersection of the concurrent sinusoidal curves by searching for the energy peak in the ρ−θ transform space. Therefore, the points in the time-frequency plane generally map to the corresponding sinusoidal curves in the ρ−θ transform space. In particular, collinear points along the straight line of GNSS sweep interference frequency modulation law in the time-frequency plane map to the corresponding concurrent sinusoidal curves intersected in the  ρ−θ transform space.

**Figure 12:**
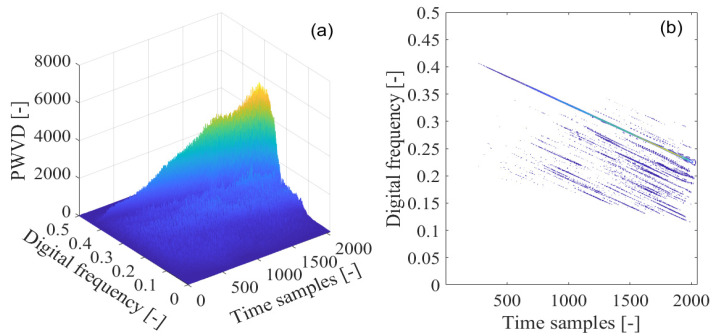
The pseudo-Wigner–Ville distribution (PWVD) of the GPS L1-C/A signal disturbed by the sweep interference; (**a**) PWVD; (**b**) PWVD’s contour.

The pseudo-Wigner–Ville distribution is adopted to mitigate the cross-interfering term present in the Wigner–Ville distribution in the detection of GNSS interference. The pseudo-Wigner–Ville distribution of the disturbed GPS L1-C/A signal is shown in Figure 12a, with the corresponding contour of the calculated pseudo-Wigner–Ville distribution provided in Figure 12b. It can be seen that the sweep interfering signal shows a linear behavior denoting its frequency modulation law in the time-frequency domain, and the cross-interfering terms are alleviated to some extent due to the use of the low-pass filtering window function in the pseudo-Wigner–Ville distribution. It is notable that the realized suppression of the undesired cross interfering terms within the quadratic time-frequency distributions is gained at the price of attenuating the property of the time-frequency aggregation with the pseudo-Wigner–Ville distribution.

**Figure 13:**
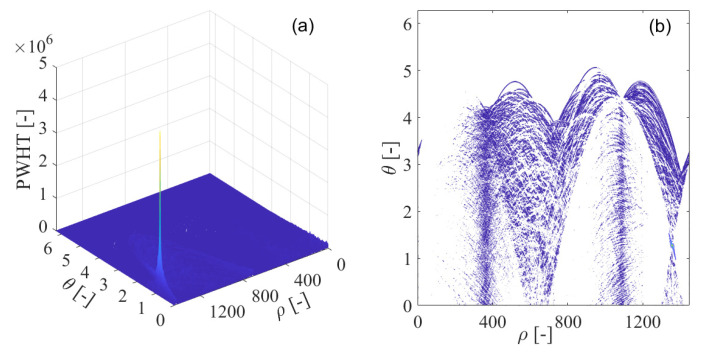
The pseudo-Wigner–Hough transform (PWHT) of GPS L1-C/A signal disturbed by the sweep interference. (**a**) PWHT; (**b**) PWHT’s contour.

In Figure 13a, the pseudo-Wigner–Hough transform is used in the detection of the sweep interfering signal, with its contour shown in Figure 13b. From the figure, the intensity of the energy peak corresponding to the sweep interference present in the received GPS L1-C/A signal can be strengthened at the intersection of the concurrent sinusoidal curves in the ρ−θ transform space, which, respectively, map to the collinear points located in the same line of GNSS sweep interference frequency modulation law in the time-frequency plane obtained by the pseudo-Wigner–Ville distribution. When the position of the intersection of the concurrent sinusoidal curves is determined in the ρ−θ transform space, the characteristic parameters of the sweep interference can be estimated. The obtained energy peak of the pseudo-Wigner–Hough transform is accumulated at the intersection since the concurrent sinusoidal curves all pass through the same point in the ρ−θ transform space. However, in the other areas excluding the intersection, the pseudo-Wigner–Hough transform energy distributions from the white Gaussian noise or the GNSS useful signal have not been accumulated. Furthermore, these energy distributions corresponding to white Gaussian noise or GNSS useful signal become sparse when compared with the case of Wigner–Hough transform, as can be seen in Figure 13b.

**Figure 14:**
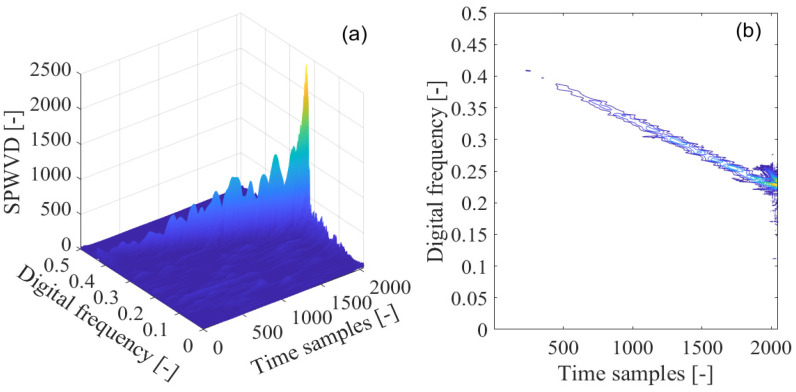
The smoothed pseudo-Wigner–Ville distribution (SPWVD) of the GPS L1-C/A signal disturbed by the sweep interference; (**a**) SPWVD; (**b**) SPWVD’s contour.

**Figure 15:**
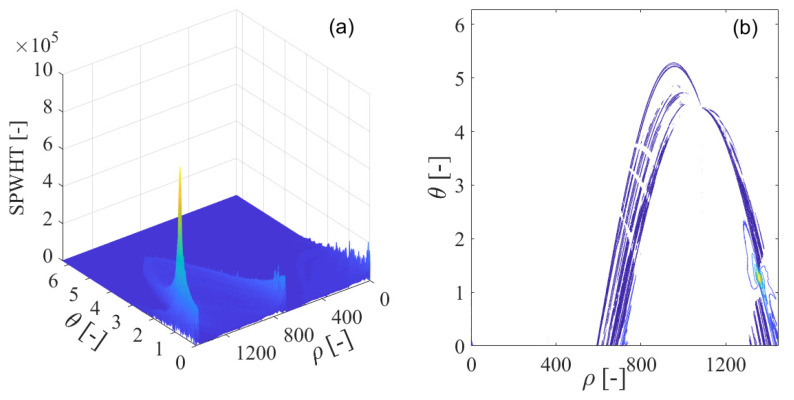
The smoothed pseudo-Wigner–Hough transform (SPWHT) of the GPS L1-C/A signal disturbed by the sweep interference; (**a**) SPWHT; (**b**) SPWHT’s contour.

To effectively deal with the problem of the cross-interfering terms present in the Wigner–Ville distribution, the smoothed pseudo-Wigner–Ville distribution is adopted for GNSS interference detection. In Figure 14a, the smoothed pseudo-Wigner–Ville distribution of the interfered GPS L1-C/A signal is shown, where the peaks of the smoothed pseudo-Wigner–Ville distribution clearly emerge in the time-frequency plane, representing the contribution from the GNSS sweep interfering signal. The corresponding smoothed pseudo-Wigner–Ville distribution’s contour is provided in Figure 14b. The undesired sweep interfering signal shows a linear characteristic in frequency denoting its frequency modulation law, which can be observed in the limited linear area within the time-frequency space. In comparison to the Wigner–Ville distribution and pseudo-Wigner–Ville distribution approaches, the cross-terms are effectively suppressed in the time-frequency plane by the smoothed pseudo-Wigner–Ville distribution through the use of two-dimensional smoothing function. However, this benefit is achieved at the cost of a deterioration in the time-frequency localization property for the analyzed signal. In Figure 15, the smoothed pseudo-Wigner–Hough transform is employed for GNSS interference detection. From Figure 15a, the intensity of the energy peak representing the sweep interference in the received GPS L1-C/A signal can be clearly observed in the intersection of the concurrent sinusoidal curves in the ρ−θ transform space, which, respectively, correspond to the collinear points distributed on the same frequency modulation line of the sweep interference in the time-frequency plane obtained by the smoothed pseudo-Wigner–Ville distribution. In comparison to the Wigner–Hough transform and pseudo-Wigner–Hough transform approaches, the smoothed pseudo-Wigner–Hough transform energy distributions contributed from the white Gaussian noise or the GNSS useful signal are much more weakened in the ρ−θ transform space, as can be seen in Figure 15b. The smoothed pseudo-Wigner–Ville distribution energy distributions have been effectively strengthened at the intersection of the corresponding concurrent sinusoidal curves in the parameter space, forming a sharp energy peak. However, in other regions, excluding the intersection in the ρ−θ transform space, the energy distributions from the white Gaussian noise or the useful GPS L1-C/A signal have not been accumulated but suppressed to very low levels even equal to zero. Therefore, the sweep interfering signal can be clearly distinguished from the others, and its signal features can be easily extracted using the proposed smoothed pseudo-Wigner–Ville distribution method.

In comparison to the global detection of many collinear points on the straight line in the time-frequency space by using the smoothed pseudo-Wigner–Ville distribution technique, the proposed smoothed pseudo-Wigner–Hough transform method easily deals with sweep interference detection since it only needs to check the local energy peak at a particular intersection within the ρ−θ transform space. The smoothed pseudo-Wigner–Hough transform-based GNSS interference detection can be implemented by the accumulator through the voting scheme in the ρ−θ transform space. Therefore, the characteristic parameters related to the sweep interference frequency modulation law in the time-frequency plane can be determined. When the position of the intersection of the concurrent sinusoidal curves is determined in the ρ−θ transform space, the characteristic parameters of the GNSS sweep interference can be then precisely estimated. This is the subject of future research.

**Figure 16:**
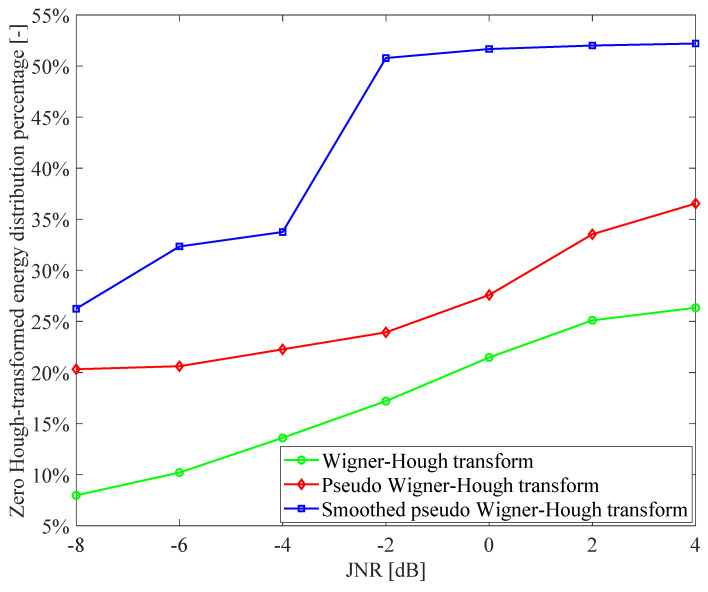
The zero Hough-transformed energy percentage metrics of the Hough transform based GNSS interference detection techniques.

Moreover, in order to further verify the GNSS interference detection performance improvement of the proposed smoothed pseudo-Wigner–Hough transform method in comparison to the other Hough transform-based techniques, the zero Hough-transformed energy percentage metrics are, respectively, evaluated for the Wigner–Hough transform, the pseudo-Wigner–Hough transform and smoothed pseudo-Wigner–Hough transform in the detection of the interfered GPS L1-C/A signal, as can be shown in Figure 16. In the zero Hough-transformed energy percentage performance analysis, the C/N_0_ of the used GPS L1-C/A signal is 46 dB-Hz, the JNR values of the sweep interference range from −8 dB to 4 dB; and the size of the local neighborhood domain in the ρ−θ parameter space is set to be an 80×80 matrix, i.e., 80×80 cells are contained in the local neighborhood domain, where the main energy peak of any Hough-transformed distribution is localized. In the evaluation of the zero Hough-transformed energy percentage for any Hough-transformed distribution in the GNSS interference detection, only the Hough-transformed energy distributions in the cells of the region within the parameter space excluding the determined local neighborhood area are considered; hence, the higher the proportion of cells with zero Hough-transformed energy distribution value in the region within the parameter space, the better performance of zero Hough-transformed energy percentage can be obtained. From Figure 16, it can be seen that as the JNR values of the interfered GPS L1-C/A signal increase, the zero Hough-transformed energy percentage results of the Hough transform-based GNSS interference detection techniques increase monotonously. This indicates that when the GNSS interference intensity increases for any type of Hough transform-based interference detection method, most of the Hough-transformed energy is more concentrated in a specific local intersection area within the parameter space, and the residual Hough-transformed energy distribution is better suppressed to zero in the other areas.

From Figure 16, it is easy to observe that the zero Hough-transformed energy percentage result obtained using the proposed smoothed pseudo-Wigner–Hough transform method is much better than the other techniques such as Wigner–Hough transform and pseudo-Wigner–Hough transform. For example, the zero Hough-transformed energy percentage result of the smoothed pseudo-Wigner–Hough transform method is at least double that of the traditional Wigner–Hough transform approach; in particular, when the JNR of the interfered GPS L1-C/A signal is lower than or equal to −4 dB, the zero Hough-transformed energy percentage of the smoothed pseudo-Wigner–Hough transform method is at least 20% higher than that of the pseudo-Wigner–Hough transform technique; in addition, when the JNR of the interfered GPS L1-C/A signal is higher than −4 dB, the zero Hough-transformed energy percentage of the smoothed pseudo-Wigner–Hough transform method is at least 42% better than that of the pseudo-Wigner–Hough transform approach. Therefore, the proposed smoothed pseudo-Wigner–Hough transform method presents superior properties of energy aggregation and cross-term suppression in the detection of GNSS interfering signal, indicating that the smoothed pseudo-Wigner–Hough transform method can provide significantly improved GNSS interference detection performance over the other Wigner–Hough transform and pseudo-Wigner–Hough transform approaches.

## 7. Discussion

From the GNSS interference detection results obtained with the Hough transform-based techniques, the proposed smoothed pseudo-Wigner–Hough transform can be successfully used to detect the GNSS sweep interference since it deals with the cross-term problem effectively and shows much-improved energy aggregation property over the pseudo-Wigner–Hough transform and Wigner–Hough transform techniques. In contrast to the traditional Wigner–Hough transform-based technique [23], the GNSS interference detection performance obtained with the smoothed pseudo-Wigner–Hough transform method is at least double that of the Wigner–Hough transform-based technique, according to the zero Hough-transformed energy percentage criteria. In contrast to the pseudo-Wigner–Hough transform-based technique [24], for any JNR value of the GNSS interfering signal, the zero Hough-transformed energy percentage obtained with the smoothed pseudo-Wigner–Hough transform-based GNSS interference detection method is improved at least 20% compared with the pseudo-Wigner–Hough transform-based technique. Therefore, the smoothed pseudo-Wigner–Hough transform method has been proven to be superior to the Wigner–Hough transform and pseudo-Wigner–Hough transform techniques in the detection of sweep interference present in the received GNSS signal.

Recently, the reassigned smoothed pseudo-Wigner–Ville distribution was combined with the Hough transform to detect GNSS interference [26]; and the rearranged wavelet-Hough transform was proposed to improve interference detection performance for GNSS receivers [16]. The reassignment algorithms implemented in the smoothed pseudo-Wigner–Ville distribution or wavelet transform cost heavy computational resources, which will cause problems in the real-time detection of GNSS interfering signals. These methods are more suitable for the anti-interference design of the FPGA-based GNSS receivers. In this paper, the smoothed pseudo-Wigner–Hough transform has been proposed in the GNSS interference detection with moderate computational complexity; this method presents better real-time performance in interference detection in comparison to the above two techniques, which is recommended for the anti-interference GNSS receiver even implemented on the low-cost software-defined radio platform.

Due to the fact that Hough transform can be used to detect arbitrary shapes and recognize complex patterns [17,18], and the nonlinear frequency modulation signal can be described by the high order polynomial function, hence, the nonlinear curve which represents the frequency modulation law for the nonlinear chirp signal can be detected and recognized using the Hough transform. Therefore, the developed Hough transform-based GNSS interference techniques are supposed to be valid and effective at dealing with nonlinear sweep interference present in the received GNSS signal. Although it is really difficult to detect the nonlinear chirp interfering signal, we will try to deal with this challenging problem of detecting nonlinear frequency modulated GNSS interference in our future research works.

## 8. Conclusions

This paper has proposed a novel smoothed pseudo-Wigner–Hough transform-based GNSS interference detection method. To demonstrate the validity and effectiveness of the proposed technique, real GPS L1-C/A data in the presence of sweep interference have been used in the GNSS interference detection tests, and full performance analysis and comprehensive comparison with the conventional time-frequency analysis-based approaches have been undertaken.

From the experimental results, it has been shown that spectrogram has a time-frequency resolution trade-off problem and Wigner–Ville distribution exhibits an undesired cross-term problem in the GNSS interference detection process, resulting in serious interference detection performance degradation. However, employing the Hough transform-based GNSS interference detection methods developed in this paper converts the original problem of global detection of the straight line of frequency modulation law of the sweep interference present in the received GNSS signal in the time-frequency space to the new problem of locally searching for the Hough-transformed energy peak positioned in a small intersecting area of concurrent sinusoidal curves in the ρ−θ transform space. As evident in the results, the developed Hough transform-based GNSS interference detection methods have been shown to be effective in the suppression of undesirable cross interfering terms present in the quadratic time-frequency distributions, and the developed methods enhance the properties of energy aggregation, facilitating improved GNSS interference detection performance over the conventional time-frequency analysis-based approaches.

Among the Hough transform-based GNSS interference detection techniques, the Wigner–Hough transform approach can be used to partially suppress the cross-terms within the quadratic time-frequency distribution; the pseudo-Wigner–Hough transform method improves interference detection performance over the Wigner–Hough transform technique; in the case of the smoothed pseudo-Wigner–Hough transform method, the Hough-transformed energy distributions are effectively strengthened and highly concentrated in a particular area of intersection within the parameter space. This enables the smoothed pseudo-Wigner–Hough transform method to be much superior to the Wigner–Hough transform and pseudo-Wigner–Hough transform techniques, as can be verified from the zero Hough-transformed energy percentage analysis results of the Hough transform-based GNSS interference detection methods.

In summary, the developed Hough transform-based GNSS interference detection methods are superior to the traditional time-frequency analysis-based approaches, and in particular, the proposed novel smoothed pseudo-Wigner–Hough transform method provides supreme interference detection performance among the developed Hough transform-based techniques. The proposed smoothed pseudo-Wigner–Hough transform method is with moderate computational complexity, which is very promising to be effectively used in the practical interference detection design for GNSS receivers in interfering environments.

## Figures and Tables

**Table 1:** Setup parameters in the GNSS interference detection test.

Parameter	Value
Sampling frequency, $f_{s}$	24 MHz
Intermediate frequency, $f_{IF}$	40.42 MHz
Code length	1023 chips
Sweep Period	0.5 ms
Analysis window	Hamming

## Data Availability

Not applicable.
